# Emerging field: *O*-GlcNAcylation in ferroptosis

**DOI:** 10.3389/fmolb.2023.1203269

**Published:** 2023-05-11

**Authors:** Hongshuo Zhang, Juan Zhang, Haojie Dong, Ying Kong, Youfei Guan

**Affiliations:** ^1^ Advanced Institute for Medical Sciences, Dalian Medical University, Dalian, China; ^2^ Department of Biochemistry and Molecular Biology, College of Basic Medical Sciences, Dalian Medical University, Dalian, China

**Keywords:** ferroptosis, *O*-GlcNAcylation, ROS biology, iron metabolism, lipid peroxidation, subcellular organelle

## Abstract

In 2012, researchers proposed a non-apoptotic, iron-dependent form of cell death caused by lipid peroxidation called ferroptosis. During the past decade, a comprehensive understanding of ferroptosis has emerged. Ferroptosis is closely associated with the tumor microenvironment, cancer, immunity, aging, and tissue damage. Its mechanism is precisely regulated at the epigenetic, transcriptional, and post-translational levels. *O*-GlcNAc modification (*O*-GlcNAcylation) is one of the post-translational modifications of proteins. Cells can modulate cell survival in response to stress stimuli, including apoptosis, necrosis, and autophagy, through adaptive regulation by *O*-GlcNAcylation. However, the function and mechanism of these modifications in regulating ferroptosis are only beginning to be understood. Here, we review the relevant literature within the last 5 years and present the current understanding of the regulatory function of *O*-GlcNAcylation in ferroptosis and the potential mechanisms that may be involved, including antioxidant defense system-controlled reactive oxygen species biology, iron metabolism, and membrane lipid peroxidation metabolism. In addition to these three areas of ferroptosis research, we examine how changes in the morphology and function of subcellular organelles (e.g., mitochondria and endoplasmic reticulum) involved in *O*-GlcNAcylation may trigger and amplify ferroptosis. We have dissected the role of *O*-GlcNAcylation in regulating ferroptosis and hope that our introduction will provide a general framework for those interested in this field.

## 1 Introduction

Cell death is an inevitable and important part of life, involving a series of biological processes. Historically, cell death has been divided into three types based on morphological changes: 1) type I cell death (apoptosis) is characterized by cytoplasmic shrinkage, plasma membrane blebbing, chromatin condensation; formation of apoptotic bodies; phagocytosis of apoptotic bodies by neighboring cells or macrophages (Galluzzi et al., 2018; Santagostino et al., 2021); 2) type II cell death (autophagy) exhibits extensive cytoplasmic vacuolization and accumulation of autophagic vacuoles (autophagosomes); no chromatin condensation; the fusion of autophagosomes with lysosomes (Lin et al., 2021); 3) type III cell death (necrosis), characterized by cell swelling, loss of membrane integrity, and “spillage” of intracellular contents (Moujalled et al., 2021). In a departure from traditional thinking, the Stockwell laboratory proposed the concept of a unique form of regulated cell death driven by iron-dependent lipid peroxidation, named ferroptosis (Dixon et al., 2012). As a new cell death modality, ferroptosis studies have increased in recent years; however, our understanding of the regulatory mechanisms for ferroptosis is still incomplete. Recently, there has been increasing evidence that pathways closely related to epigenetic regulation and post-translational modification mechanisms influence the propensity of cells to undergo ferroptosis (Wei et al., 2020; Chen D. et al., 2021; Tang et al., 2021).

As one of the prevalent post-translational modifications, the O-linked β-N-acetylglucosamine (*O*-GlcNAc) modification consists of the addition of a single N-acetylglucosamine (GlcNAc) to serine or threonine residues of a protein. Unlike conventional *N*-/*O*-glycosylation, *O*-GlcNAcylation is not restricted to the extracellular structural domains of the endoplasmic reticulum (ER), Golgi apparatus, or secretory proteins (Hart, 2019). It also occurs in the cytoplasm, nuclear proteins, and mitochondria. Unlike kinases and phosphatases involved in phosphorylation, the regulation of *O*-GlcNAcylation has so far been the responsibility of only two enzymes, *O*-GlcNAc transferase (OGT) and *O*-GlcNAcase (OGA). OGT catalyzes the addition of the precursor uridine diphosphate-GlcNAc (UDP-GlcNAc) to proteins, and OGA removes GlcNAc by hydrolysis (Vocadlo, 2012). As a branch of glucose metabolism, UDP-GlcNAc is the end product of the hexosamine biosynthetic pathway (HBP), which links glucose, fatty acid, nucleic acid, and amino acid metabolic pathways. Therefore, *O*-GlcNAcylation is involved in various life processes as a nutrient sensor and signaling integrator. Our previous work showed that *O*-GlcNAcylation could redirect glucose metabolism and provide compensation for glycolysis via glycerol (Zhang H. et al., 2022). Our previous review also detailed the important role of *O*-GlcNAc modification on physiological and pathological processes such as glucose metabolism, lipid metabolism, and homeostasis maintenance (Zhang H. et al., 2021). In addition, *O*-GlcNAc modifications are highly sensitive to various stimuli, including physiological, chemical, and oxidative stress, heat shock, hypoxia, and ischemia (Zachara and Hart, 2004). Protein molecules respond to stress stimuli by transient and reversible regulation of *O*-GlcNAcylation, thereby improving the survival adaptability of cells and preventing cell death (Xue et al., 2022).

Although ferroptosis has been detected in various biological systems since 2012, understanding the role and mechanism of *O*-GlcNAcylation in regulating ferroptosis is just beginning; the first regulation study was reported in 2019 (Chen et al., 2019). We reviewed the relevant literature within the last 5 years. Here, we present the role of *O*-GlcNAcylation in regulating ferroptosis from the perspectives of antioxidant defense system-controlled ROS biology, iron metabolism, lipid metabolism and peroxidation, and the morphology and function of subcellular organelles (mitochondria, ER). We also describe the potential mechanisms that may be involved. We hope our review provides a general framework for those interested in this field.

## 2 Overview of ferroptosis

**FIGURE 1:**
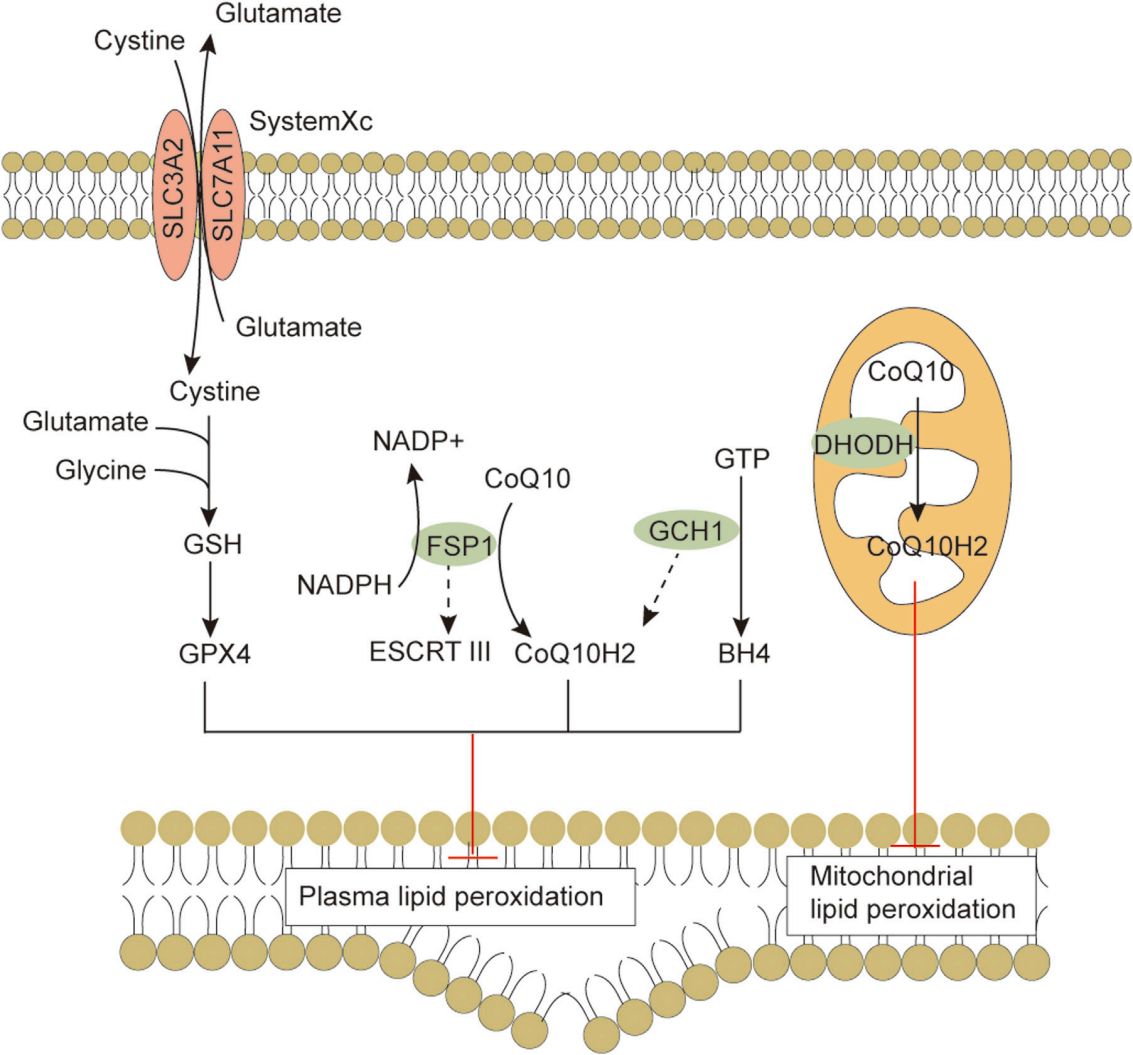
The antioxidant defense systems in ferroptosis. Cells have evolved at least four ferroptosis antioxidant defense systems mainly including the System Xc/GSH/GPX4 system, the FSP1/CoQ10H2 system, the GCH1/BH4 system and the DHODH/CoQH2 system. When the detoxification capacity provided by the cellular ROS defense system is insufficient, membrane lipid peroxidation leads to subsequent ferroptosis. Abbreviations: BH4, tetrahydrobiopterin; CoQ10, coenzyme Q10; CoQ10H2, ubiquinol; DHODH, dihydroorotate dehydrogenase; ESCRTⅢ, endosomal sorting complex required for transport III; FSP1, ferroptosis suppressor protein 1; GCH1, GTP cyclohydrolase 1; GPX4, Glutathione peroxidase 4; GSH, glutathione; SLC3A2, solute carrier family 3 member 2; SLC7A11, solute carrier family 7 member 11.

**FIGURE 2:**
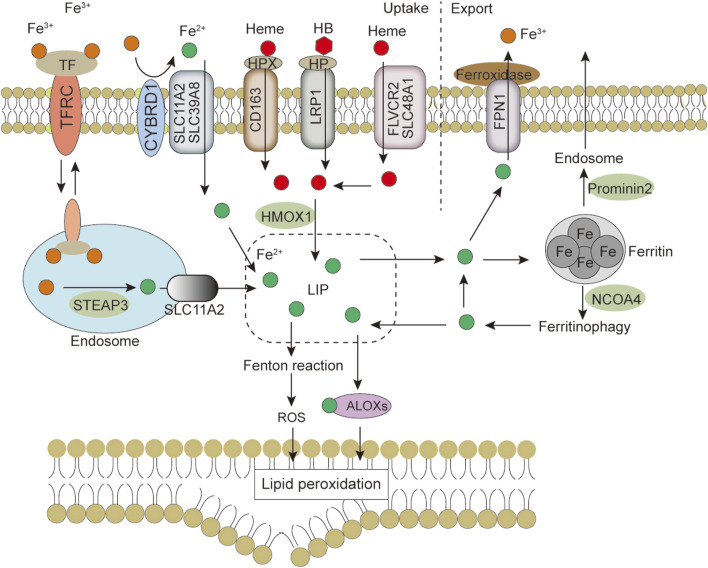
Iron metabolism and ferroptosis. Iron import is mainly mediated by the TF-TFRC, NTBI and heme pathways, intracellular iron is utilized from LIP, excess iron is stored back in ferritin, and iron is exported extracellularly via the FNP1 or exosome pathway. Changes in iron homeostasis regulate cell sensitivity to ferroptosis. Abbreviations: ALOXs, arachidonate lipoxygenase; CYBRD1, cytochrome B reductase 1; FLVCR2, feline leukemia virus subgroup C cellular receptor 2; FPN1, ferroportin 1; HB, hemoglobin; HMOX1, heme oxygenase 1; HP, haptoglobin; HPX, hemopexin; LIP, labile iron pool; LRP1, LDL receptor-related protein 1 receptor; NCOA4, nuclear receptor coactivator 4; ROS, reactive oxygen species; SLC11A2, solute carrier family 11 member 2; SLC39A8, solute carrier family 39 member 8; SLC48A1, solute carrier family 48 member 1; STEAP3, six-transmembrane epithelial antigen of the prostate 3; TF, transferrin; TFRC, transferrin receptor.

**FIGURE 3:**
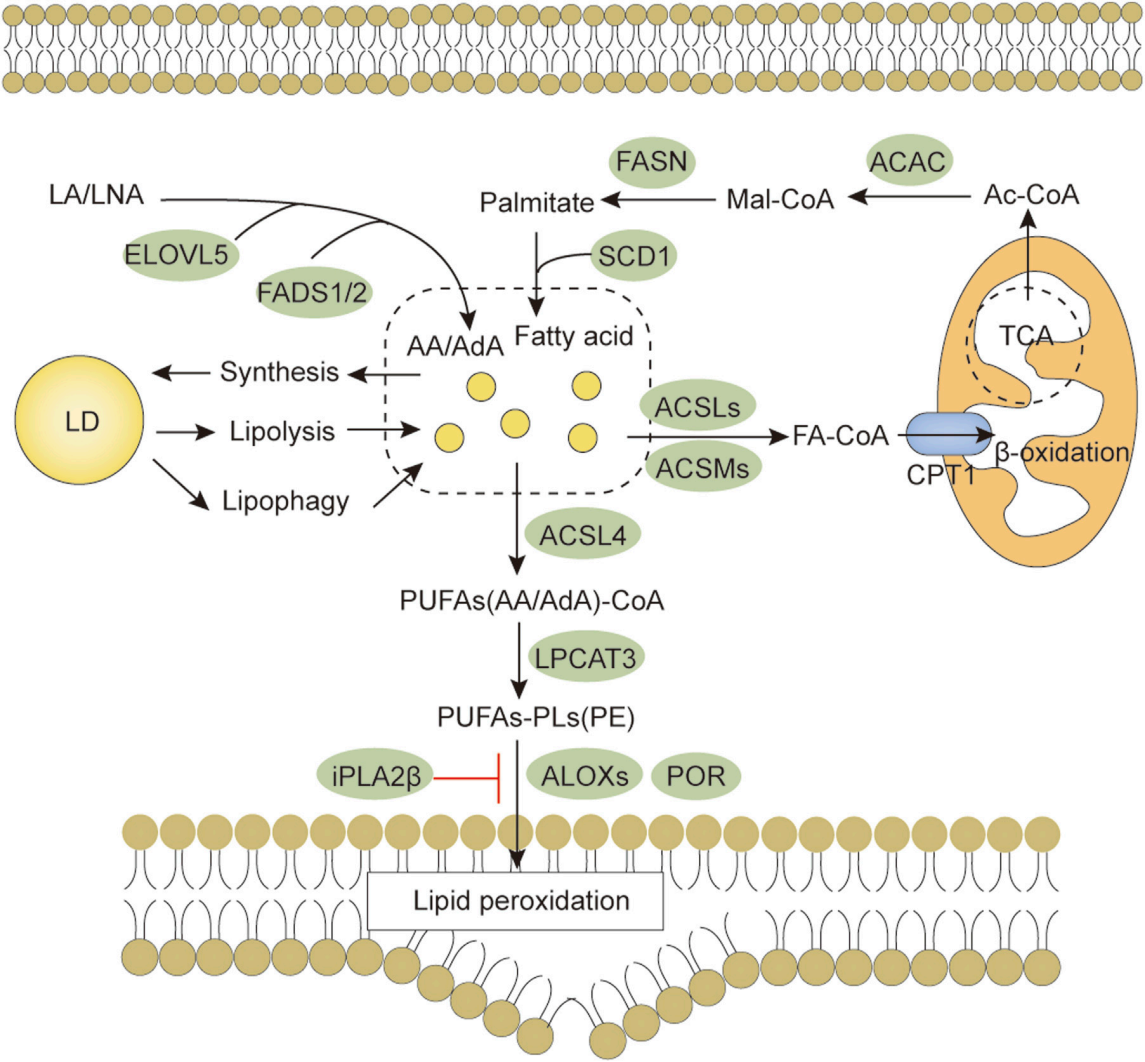
Lipid metabolism and ferroptosis. Both lipid anabolism and catabolism affect the content of lipid substrates for ferroptosis. PUFAs are activated by ACSL4, LPCAT3 promotes the synthesis of PUFAs-PL, and membrane PUFAs-PL are subsequently oxidized by oxygenases, such as ALOX and POR, to promote ferroptosis. Abbreviations: AA, arachidonic acid; ACAC, acetyl-CoA carboxylase; ACSLs, Long-chain fatty acid-CoA synthases; ACSMs, medium-chain acyl-CoA synthases; AdA, adrenic acid; ALOXs, arachidonate lipoxygenase; CPT1, carnitine palmitoyltransferase 1; LA, linoleic acid; LD, lipid droplet; LNA, α-linolenic acid; ELOVL5, elongation of very long-chain fatty acid protein 5; FADS1/2, fatty acid desaturases 1/2; FASN, fatty acid synthetase; LPCAT3, lysophosphatidylcholine acyltransferase 3; POR, Cytochrome P450 reductase.

**FIGURE 4:**
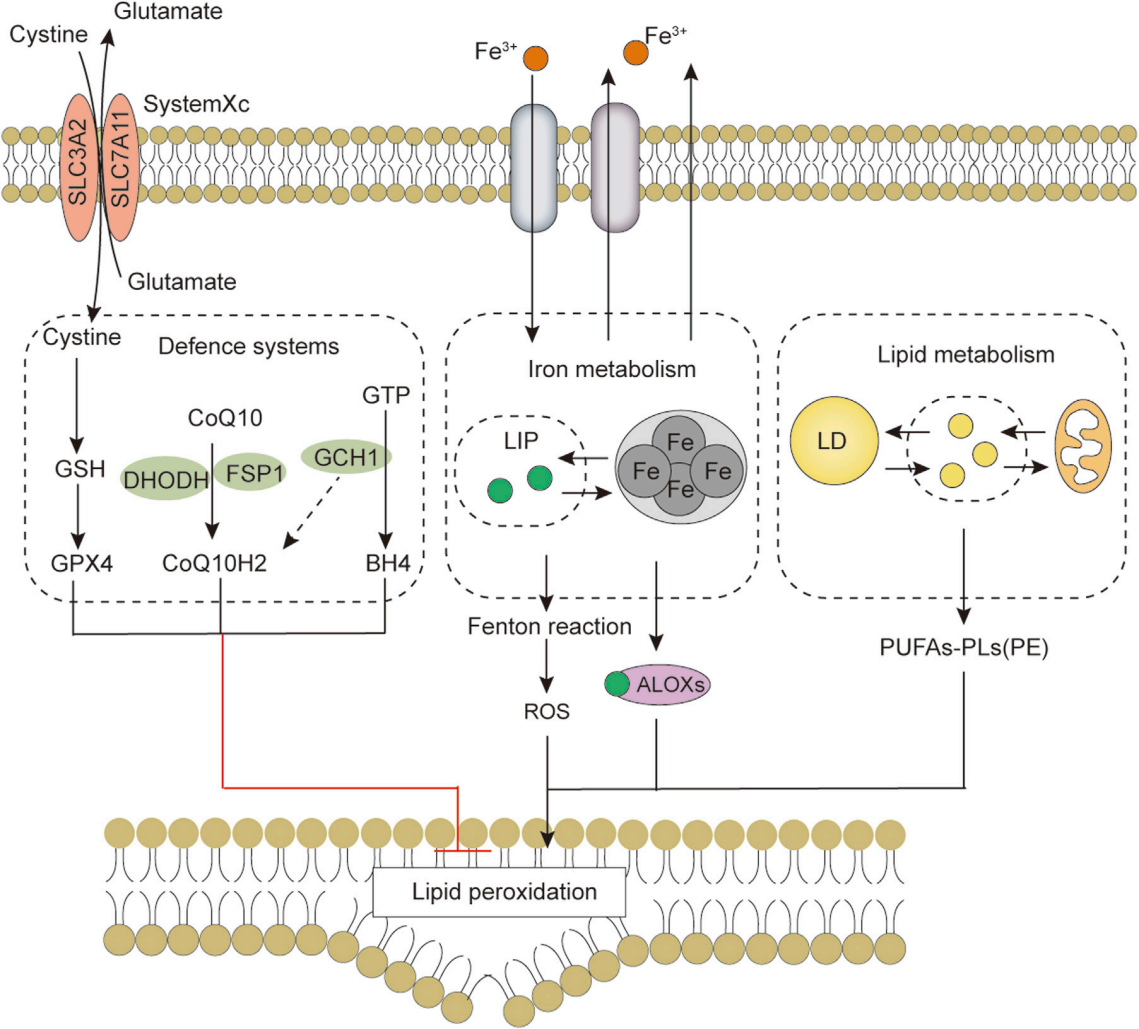
The core features of ferroptosis include the antioxidant defense system, iron metabolism, lipid metabolism and peroxidation. Abbreviations: ALOXs, arachidonate lipoxygenase; BH4, tetrahydrobiopterin; CoQ10, coenzyme Q10; CoQ10H2, ubiquinol; DHODH, dihydroorotate dehydrogenase; FSP1, ferroptosis suppressor protein 1; GCH1, GTP cyclohydrolase 1; GPX4, Glutathione peroxidase 4; GSH, glutathione; LD, lipid droplet; LIP, labile iron pool; PUFAs, Free polyunsaturated fatty acids; PUFAs-PL, PUFA-phospholipids; PUFAs-PE, PUFA-phosphatidylethanolamine; ROS, reactive oxygen species; SLC3A2, solute carrier family 3 member 2; SLC7A11, solute carrier family 7 member 11.

Ferroptosis is morphologically, functionally, and biochemically distinct from other forms of cell death, such as apoptosis, necrosis, and autophagy (Dixon et al., 2012; Xie et al., 2016). Morphologically, ferroptosis is mainly characterized by abnormalities in mitochondria, including a marked shrinkage of mitochondrial volume, increased bilayer density, reduction or disappearance of mitochondrial cristae, and rupture of the outer membrane (Li et al., 2020). In some cases, ferroptosis is accompanied by an increase in intracellular autophagosomes and cell detachment and aggregation (Yagoda et al., 2007; Friedmann Angeli et al., 2014). Moreover, ferroptosis occurring in one cell can rapidly spread to neighboring cells (Katikaneni et al., 2020; Riegman et al., 2020). Genetically, mutations or polymorphisms in some genes (e.g., P53 and RAS) are involved in the biological regulation of ferroptosis (Jiang et al., 2015). Biochemically, there are three main areas of research regarding the regulation of ferroptosis, including antioxidant defense system-controlled ROS biology (Figure 1), iron metabolism (Figure 2), and membrane lipid peroxidation metabolism (Figures 3, 4). The accumulation of reactive oxygen species (ROS) due to the inactivation of the antioxidant defense system seems to play a key role in ferroptosis; however, not all sources of ROS contribute equally to ferroptosis. Iron-dependent ROS production appears to be the main driver of ferroptosis induced by lipid peroxidation. The antioxidant defense system of ferroptosis consists of the classical System Xc/GSH/GPX4 pathway. Cystine (Cys2) and glutamate (Glu) enter and exit cells via System Xc in a 1:1 reverse transport, and the absorbed Cys2 can be oxidized to cysteine (Cys) for the synthesis of glutathione (GSH). Glutathione peroxidase 4 (GPX4) uses glutathione as a cofactor to reduce lipid peroxides to lipid alcohols. Inhibition of System Xc or reduced GPX4 activity leads to lipid ROS accumulation, promoting ferroptosis (Friedmann Angeli et al., 2014; Chen et al., 2021b). Several other GPX4-independent ferroptosis suppressor systems have been identified in recent years [e.g., the ferroptosis suppressor protein 1 (FSP1)/CoQ_10_H_2_ system, GTP cyclohydrolase 1 (GCH1)/tetrahydrobiopterin (BH4) system, and dihydroorotate dehydrogenase (DHODH) system] (Figure 1). The FSP1 (also known as apoptosis-inducing factor mitochondria-associated 2, AIFM2) oxidoreductase of the FSP1/CoQ_10_H_2_ system reduces ubiquinone (CoQ, common CoQ_10_) to ubiquinol (CoQ_10_H_2_) using NADPH (Bersuker et al., 2019; Doll et al., 2019). CoQ_10_H_2_ can capture peroxyl radicals to scavenge lipid peroxidation intermediates (Bersuker et al., 2019). Furthermore, FSP1 participates in the repair of plasma membrane damage by activating the endosomal sorting complex required for transport III(ESCRT III) (Dai et al., 2020). In the GCH1/BH4 system, GCH1 produces the lipophilic antioxidant BH4, which functions similarly to CoQ_10_H_2_ to prevent lipid peroxidation and inhibit ferroptosis. GCH1 also causes remodeling of the lipid membrane environment to increase CoQ_10_H_2_ levels and deplete polyunsaturated fatty acid-phospholipids (PUFA-PLs), reducing ferroptosis sensitivity (Kraft et al., 2020; Soula et al., 2020). DHODH was identified in 2021 as a local mitochondrial defense system. When GPX4 is acutely inhibited, DHODH increases the flux of CoQ_10_ reduction to CoQ_10_H_2_, acting as an iron death inhibitor (Mao et al., 2021). Recent studies suggest that there may be other defense systems. In 2021, Zeitler et al. found that the metabolite indole-3-pyruvate (In3Py) could inhibit iron death by scavenging free radicals and attenuating ferroptosis-related gene expression (Zeitler et al., 2021).

Some iron regulatory-related proteins, including transferrin (TF), transferrin receptor (TFRC), and ferritin components (FTH1 and FTL), affect intracellular Fe^2+^ levels by regulating iron metabolism. Fe^2+^ oxidizes lipids in a Fenton-like manner, generating large amounts of ROS and promoting ferroptosis (Chen et al., 2021c). Free polyunsaturated fatty acids (PUFAs) generated by lipid anabolism and catabolism are sensitive to lipid peroxidation (Yang and Stockwell, 2016). Long-chain fatty acid-CoA synthetase 4 (ACSL4) promotes the activation of PUFAs (especially arachidonic acid and adrenergic acids) into PUFAs-CoA, lysophosphatidylcholine acyltransferase 3 (LPCAT3) promotes the binding of PUFAs-CoA to phospholipids to form membrane phospholipids and thus transmit ferroptosis signaling. Downregulation of ACSL4 and LPCAT3 reduces the accumulation of membrane phospholipid peroxide substrates, which inhibits ferroptosis (Kagan et al., 2017). These are the main biochemical features of ferroptosis.

## 3 *O*-GlcNAcylation regulates cell death

Numerous studies have demonstrated that *O*-GlcNAc modifications are involved in regulating cell death. For example, decreased *O*-GlcNAcylation caused by OGT inhibitor OSMI-1 enhances doxorubicin-induced apoptosis in hepatocellular carcinoma (HCC) cells (Lee and Kwon, 2020). Glucosamine enhances *O*-GlcNAc signaling and attenuates apoptosis in iohexanol-induced renal injury (Hu et al., 2017). Melatonin inhibits bladder cancer cell proliferation and promotes apoptosis by inhibiting *O*-GlcNAcylation (Wu et al., 2021). OGT deletion-mediated downregulation of *O*-GlcNAc modification levels causes excessive hepatocyte necrosis (Zhang et al., 2019). Sevoflurane postconditioning-induced enhancement of *O*-GlcNAcylation of RIPK3 (necrosis regulatory protein) inhibits myocardial ischemia-reperfusion injury-mediated necrosis through inhibition of RIPK3/MLKL (a key mediator of necrosis) complex formation (Zhang et al., 2020). In addition, multiple mechanisms are likely involved in regulating cellular autophagy through *O*-GlcNAc signaling (Fahie and Zachara, 2016). For instance, *O*-GlcNAcylation can regulate autophagy by modifying CopII, a protein associated with autophagic vesicles (Dudognon et al., 2004). In HPV-infected head and neck squamous cell carcinomas, *O*-GlcNAc signaling elevates autophagy levels by regulating the autophagy-initiating enzyme ULK1 (Shi et al., 2022). Decreased *O*-GlcNAcylation levels in SNAP-29 induced by OGT downregulation promote the formation of autophagosomes and increase cisplatin resistance in ovarian cancer cells (Zhou et al., 2018). Although the mechanisms by which *O*-GlcNAcylation regulates cell death are slowly being revealed, the roles these modifications play under similar stimulation conditions may not be identical due to the effect of the spatiotemporal specificity of *O*-GlcNAc-modified proteins on the interactions between proteins and the regulatory complexity of site-specificity on altered protein function.

## 4 *O*-GlcNAcylation regulates antioxidant defense system-controlled ROS biology during ferroptosis

### 4.1 *O*-GlcNAcylation and ROS biology

Cells often encounter ROS-mediated stress. ROS act as signaling molecules and disrupt proteins, nucleic acids, and lipids, triggering cellular stress responses (Panieri and Santoro, 2016; Chen et al., 2018). Reports suggest a link between *O*-GlcNAcylation and ROS biology (Yang et al., 2010; Chen et al., 2018). For example, hydrogen peroxide enhances *O*-GlcNAcylation in mouse embryonic fibroblasts, which may be a strategy to promote cell survival in response to oxidative stress stimuli (Lee et al., 2016). Interestingly, modulation of *O*-GlcNAcylation can also affect the level of intracellular ROS. Decreased *O*-GlcNAc modifications inhibit high glucose-stimulated ROS production in rat mesangial cells (Goldberg et al., 2011). In addition, *O*-GlcNAcylation of phosphofructokinase 1 (PFK1, a key glycolytic enzyme) shifts glucose flux from glycolysis to the pentose phosphate pathway (PPP), leading to increased levels of NADPH and GSH and reduced accumulation of ROS in cancer cells (Yi et al., 2012). Similarly, glucose-6-phosphate dehydrogenase (G6PD, a rate-limiting enzyme of the PPP) activity and oligomerization are regulated by *O*-GlcNAcylation, and increased *O*-GlcNAcylation leads to dimeric G6PD accumulation, leading to increased PPP flux and NADPH and GSH levels and decreased ROS levels (Rao et al., 2015). This evidence demonstrates that the crosstalk between *O*-GlcNAcylation and ROS signaling is complex and reciprocal. Since ROS accumulation (especially inactivation of the System Xc/GSH/GPX4-dependent antioxidant defense system) is one of the essential conditions for ferroptosis, the crosstalk between abnormal ROS signaling and *O*-GlcNAc modifications may be related to ferroptosis.

### 4.2 *O*-GlcNAcylation and antioxidant defense systems

In 2019, Chen et al. (Chen et al., 2019) reported the first study on *O*-GlcNAcylation and the regulation of ferroptosis. They found that overall *O*-GlcNAc modification levels were inhibited in hepatoma cells after erastin treatment. Erastin inhibited the binding of c-Jun to OGT. Moreover, *O*-GlcNAcylated c-Jun inhibited ferroptosis by directly binding to the promoters of key enzymes in the GSH synthesis pathway [i.e., phosphoserine aminotransferase 1 (PSAT1) and cystathionine β synthase (CBS)], activating their transcription, stimulating GSH synthesis, and decreasing ROS accumulation (Chen et al., 2019). This study opened the door to understanding the role of *O*-GlcNAc modifications in iron-dependent death (i.e., ferroptosis). However, for the classical ferroptosis antioxidant system (System Xc/GSH/GPX4 axis), System Xc and GPX4 may also be potential targets for regulation by *O*-GlcNAcylation. System Xc is an amino acid reverse transporter protein in the membrane phospholipid bilayer, consisting of two subunits, solute carrier family 7 member 11 (SLC7A11) and solute carrier family 3 member 2 (SLC3A2). The expression and activity of SLC7A11 affect cystine uptake by System Xc. They are positively regulated by the oxidative stress regulator nuclear factor erythroid 2-related factor 2 (NRF2) (Chen et al., 2017) and negatively regulated by tumor suppressor genes, such as P53 (Jiang et al., 2015), BAP1 (Zhang et al., 2018), and BECN1(Song et al., 2018). However, one study found that the OGA inhibitor Thiamet G (TMG) decreased NRF2 protein and transcript levels but increased the *O*-GlcNAcylation of NRF2(Tan et al., 2017). P53 activity is strictly regulated, and post-translational modifications are an important dimension of this regulation. P53 is modified by *O*-GlcNAc; *O*-GlcNAcylation of Ser149 stabilizes P53 by blocking ubiquitin-dependent protein degradation (Yang et al., 2006). Elevated *O*-GlcNAc modifications induced by TMG promote the activation and nuclear localization of P53 (de Queiroz et al., 2016). Furthermore, BAP1 and BECN1 can also be modified by *O*-GlcNAc(Wang and Hanover, 2013; Moon et al., 2017). These examples suggest that *O*-GlcNAcylation indirectly regulate System Xc and may play a regulatory role in ferroptosis.

GPX4 directly reduces cytotoxic lipid peroxides (L-OOH) to non-toxic lipid alcohols (L-OH) in the membrane. Of the three GPX4 isoforms (mitochondrial, nuclear, and cytoplasmic isoforms), only the cytoplasmic isoform is required for ferroptosis, and its expression is regulated by transcription factors (i.e., stimulating protein 1, nuclear factor Y, and AP2) (Ursini and Maiorino, 2020; Zheng and Conrad, 2020), integrin α6β4 (Brown et al., 2017), and heat shock protein 90 (Wu et al., 2019). It is not clear whether these molecules undergo *O*-GlcNAcylation and regulate GPX4. *O*-GlcNAcylation is mainly in the cytoplasm and nucleus, while GPX4 is localized in the cytoplasm, mitochondria, and mitochondrial OGT has also been identified in recent years (Hanover et al., 2003). In addition, we used the *O*-GlcNAc modification site prediction website (https://services.healthtech.dtu.dk/service.php?YinOYang-1.2) for GPX4 and showed the highest potential at 67 threonine (NetPhos threshold>0.5), which implies that GPX4 may directly modified by *O*-GlcNAc. The above evidence implies that *O*-GlcNAc signaling homeostasis may affect ferroptosis sensitivity by regulating antioxidant defense system-controlled ROS biology through related molecules or direct modifications by transcription-dependent and -independent mechanisms.

When System Xc transfers Cys2 into cells, it also carries endogenous glutamate out of the cells. Therefore, inhibition of System Xc reduces Cys2 uptake while increasing endogenous glutamate accumulation, suggesting that intracellular glutamate metabolism may also be involved in regulating ferroptosis. In 2021, Zhang et al. investigated the mechanism that determines the sensitivity of lung adenocarcinoma cells to ferroptosis (Zhang X. et al., 2021) and found that the greater the inhibition of ferroptosis by erastin, the lower the Yes-associated protein (YAP) levels. Erastin induces polyubiquitination-mediated degradation of YAP by recruiting the ubiquitin E3 ligase βtrCP. However, RSL3, a ferroptosis stimulator targeting GPX4, failed to reduce YAP levels, suggesting that the reduction of YAP was caused by the inhibition of Systemic Xc. It was further found that cystine deficiency caused by System Xc inhibition is critical for triggering ferroptosis. Moreover, glutamate accumulation is required to determine ferroptosis sensitivity after System Xc inhibition, and the decrease in YAP is caused by endogenous glutamate accumulation. Phosphorylation of YAP S127 (the major phosphorylation site of the Hippo pathway) is increased by erastin in a glutamate-dependent manner, whereas the *O*-GlcNAcylation on T241 of YAP and total YAP are significantly decreased by this treatment (Zhang H. et al., 2021). In addition, there is evidence that the *O*-GlcNAcylation at T241 stabilizes YAP by antagonizing Hippo-dependent phosphorylation (Zhang et al., 2017). Decreased *O*-GlcNAcylation is a prerequisite for endogenous glutamate-induced reduction of YAP levels, and *O*-GlcNAcylation at T241 is critical for ferroptosis sensitivity (Zhang X. et al., 2021). However, there are no reports on *O*-GlcNAcylation and the three other antioxidant defense systems of ferroptosis independent of the System Xc/GSH/GPX4 axis. There may be synergistic or complementary effects among these antioxidant defense systems, and whether *O*-GlcNAcylation is involved in the direct or indirect regulation of these pathways or affect ferroptosis needs further investigation.

## 5 *O*-GlcNAcylation regulates iron metabolism during ferroptosis

### 5.1 *O*-GlcNAcylation and iron uptake

As an indispensable trace element in the human body, iron is essential in physiological concentrations for metabolic processes, such as oxygen transport and electron transfer. Iron promotes lipid peroxidation during ferroptosis by at least two mechanisms. Iron can produce ROS through the Fenton-like reaction and can also act as a cofactor to activate iron-containing enzymes [e.g., arachidonate lipoxygenase (ALOX) (Yang et al., 2016), Cytochrome P450 reductase (POR) (Koppula et al., 2021)] involved in lipid peroxidation. Thus, the regulation of iron metabolism (iron uptake, utilization, storage, efflux) (Figure 2) affects ferroptosis sensitivity (Chen P. H. et al., 2020).

Iron uptake into cells occurs by multiple mechanisms. The main mechanism of iron uptake is through the TF-bound iron uptake pathway (Ruff and Whittlesey, 1993; Wang J. et al., 2020; Chen D. et al., 2021). TF can bind two Fe^3+^ and subsequently bind to the TFRC, which causes membrane invagination to form specialized endosomes. The pH drop in the endosomes leads to the release of Fe^3+^ from TF. The metalloreductase six-transmembrane epithelial antigen of the prostate 3 (STEAP3) can reduce the released Fe^3+^ to Fe^2+^, which then crosses the endosomal membrane via solute carrier family 11 member 2 (SLC11A2) into the cytoplasm. TF/TFRC then returns to the cell surface (Chen et al., 2021b). It is now generally accepted that TF has a limited transport capacity and that excess iron may enter the cytosol by other routes. One such route is the non-TF-bound iron (NTBI) uptake pathway. Iron reductase or the release of cellular reductants (e.g., cytochrome B reductase 1 [CYBRD1], ascorbate) (Lane et al., 2015) present on the cell surface can reduce iron to the ferrous form and translocate it into the cell via transmembrane transport proteins, such as SLC11A2, SLC39A8 or SLC39A14 (Knutson, 2019). The Heme and hemoglobin (HB) iron uptake pathway is another mechanism of iron transport into cells. Plasma-free heme and HB are captured by hemopexin (HPX) and haptoglobin (HP) and transferred into cells by binding to the LDL receptor-related protein 1 receptor (LRP1) and macrophage scavenger receptor CD163, respectively (Gozzelino and Soares, 2014). Finally, extracellular albumin-bound heme or non-protein-bound heme enters cells via multiple transporters [feline leukemia virus subgroup C cellular receptor 2 (FLVCR2), SLC48A1, and SLC46A1] and subsequently releases Fe^2+^ through the action of heme oxygenase 1 (HMOX1) (Knutson, 2019). Iron uptake-mediated iron metabolism is essential for ferroptosis, and there is evidence that TFRC knockdown prevents ferroptosis caused by erastin or Cys2 deprivation (Yang and Stockwell, 2008; Gao et al., 2015). Therefore, does *O*-GlcNAcylation regulate ferroptosis through iron uptake? Zhu et al. (2021) showed that *O*-GlcNAcylation increased RSL3-induced ferroptosis in HCC cells via YAP, and the OGA inhibitor PUGNAc-induced *O*-GlcNAcylation promoted YAP expression and nuclear localization (Zhu et al., 2021). YAP can directly bind to the TFRC promoter region and increase TFRC expression, promoting cellular iron uptake (Torii et al., 2016). These findings suggest that *O*-GlcNAcylation can affect ferroptosis by modulating TFRC and enhancing cellular iron uptake.

### 5.2 *O*-GlcNAcylation and iron storage

Fe^2+^ released into the cytoplasm enters the metabolically active “labile iron pool” (LIP), which regulates the Fe^2+^ ion concentration in the cytoplasm and determines the exchange and utilization of Fe^2+^. Excess iron is mainly stored in cytoplasmic ferritin, a complex composed of two isoforms, ferritin heavy chain (FTH1) and ferritin light chain (FTL). FTH1 is responsible for the oxidation of Fe^2+^ to Fe^3+^, and FTL promotes iron nucleation and mineralization. Iron entering the inner cavity of ferritin is deposited as ferrihydrite (Chen et al., 2021c). Iron can be excreted through pores on the surface of ferritin; however, it is mainly bound to nuclear receptor coactivator 4 (NCOA4) and delivered to the lysosome for degradation (ferritinophagy), releasing stored iron (Mancias et al., 2014). Iron can also be released through ferritin degradation by the ubiquitin-proteasome system (De Domenico et al., 2006). Reduced FTH1 expression increases free Fe^2+^ in the LIP, promoting cellular ferroptosis (Chen X. et al., 2020). Increased iron storage by inhibiting NCOA4-mediated ferritinophagy limits ferroptosis in cancer cells (Hou et al., 2016). There is indirect evidence that *O*-GlcNAcylation regulates iron storage. First, reduced *O*-GlcNAcylation of YAP induced by endogenous glutamine accumulation in erastin-treated lung adenocarcinoma cells caused YAP degradation, and inhibition of YAP could not increase FTH1 expression via the transcription factor TFCP2, leading to elevated labile iron (Zhang H. et al., 2021). Second, Yu et al. (2022) showed that RSL3 caused biphasic changes in protein *O*-GlcNAcylation, which regulates ferroptosis by coordinating ferritinophagy and mitophagy (Yu et al., 2022). Specifically, *O*-GlcNAcylation increased sharply after RSL3 treatment and then gradually decreased during ferroptosis. Decreased *O*-GlcNAcylation strongly promoted RSL3-induced TFRC membrane transfer and increased ferroptosis sensitivity. Decreased *O*-GlcNAcylation also promoted FTH1 degradation and ferritinophagy, leading to increased labile iron levels. NCOA4 knockdown blocked ferritin co-localization with lysosomes. In contrast, de-*O*-GlcNAcylation of FTH1 at S179 increased its interaction with NCOA4 and promoted the transport of FTH1 to lysosomes, leading to ferritinophagy (Yu et al., 2022). This recent study demonstrated that *O*-GlcNAc modifications could affect ferroptosis by regulating iron storage.

### 5.3 *O*-GlcNAcylation and iron export

Iron export is mediated by the plasma membrane protein ferroportin 1 (FPN1, also known as solute carrier family 40 member 1, SLC40A1) and the ferro-oxidases (e.g., ceruloplasmin and hephaestin). FPN1 transports Fe^2+^ to the extracellular space, where it is oxidized by the ferro-oxidases to Fe^3+^ for export. In addition, ferritin and its stored iron can be released from the cell via the prominin-2-mediated exosome pathway (Brown et al., 2019), suggesting that intracellular iron export is also regulated by this secretory pathway. There is evidence that blocking the iron release pathway by inhibiting FPN1 on the cell membrane increases ferroptosis sensitivity (Brown et al., 2019; Shang et al., 2020). Moreover, NH_4_Cl can increase *O*-GlcNAcylation and FPN1 expression (Görg et al., 2019), suggesting a potential link between *O*-GlcNAcylation and iron export. However, the specific mechanism is still unclear.

## 6 *O*-GlcNAcylation regulates lipid metabolism and peroxidation during ferroptosis

Ferroptosis is ultimately caused by the peroxidation of membrane lipids and involves complex lipid metabolic processes (Figure 3). Because the bis-allylic group (-CH = CH-CH2-CH = CH-) is more susceptible to oxidation, PUFAs are one of the main lipid targets for peroxidation. Fatty acids are important precursors of membrane lipids. For their *de novo* synthesis, acetyl-CoA carboxylase (ACAC) catalyzes the synthesis of malonyl-CoA from acetyl CoA, and then fatty acid synthetase (FASN) catalyzes the condensation of malonyl-CoA and acetyl CoA to form saturated fatty acids (e.g., palmitic acid). The saturated fatty acids are desaturated by desaturases (e.g., stearoyl-CoA desaturase-1, SCD1) to form monounsaturated fatty acids (e.g., palmitoleic acid). Essential PUFAs [α-linolenic acid (LNA) and linoleic acid (LA)] obtained from food are further processed by elongation reactions (e.g., elongation of very long-chain fatty acid protein 5, ELOVL5) and desaturation (e.g., fatty acid desaturases 1/2, FADS1/2) to other long-chain PUFAs (e.g., arachidonic acid). Inhibition of ACAC can inhibit ferroptosis caused by various stimuli (Lee H. et al., 2020). SCD1 knockdown sensitizes cells to ferroptosis (Tesfay et al., 2019). FADS1/2 and ELOVL5 can promote ferroptosis (Lee J. Y. et al., 2020), suggesting that PUFA synthesis also regulates ferroptosis. *O*-GlcNAc modifications have been associated with fatty acid synthesis. For example, Sodi et al. (2018) found that OGT downregulation inhibited fatty acid biosynthesis and led to cancer cell death by decreasing FASN expression (Sodi et al., 2018). However, there is still a lack of reports on whether *O*-GlcNAcylation affects ferroptosis by regulating fatty acid synthesis. Recently, Wang et al. (2022) found enhanced *O*-GlCNAcylation at Ser555 of transcription factor ZEB1 in mesenchymal pancreatic cancer cells, which promoted FASN and FADS2 transcriptional activity and lipid peroxidation-dependent ferroptosis in cancer cells (Wang et al., 2022). This study may be the only direct evidence so far that elevated *O*-GlcNAcylation regulates ferroptosis through lipid metabolism. Interestingly, FASN is modified by *O*-GlcNAc (Baldini et al., 2016). Whether elevated *O*-GlcNAcylation plays a role in regulating ferroptosis through FASN remains to be proven.

Excess lipids can be stored in lipid droplets composed of glycerol and cholesterol lipids. Lipid droplet formation increases fatty acid storage, and separating PUFA from membrane phospholipids limits ferroptosis precursor utilization (Bai et al., 2019). Enhanced selective autophagic degradation of lipid droplets increases the production of free fatty acids, promoting lipid peroxidation and increasing the ferroptosis sensitivity of HCC cells (Bai et al., 2019). Both anabolism and β-oxidative catabolism first require fatty acid activation. Fatty acids are converted to medium/long-chain acyl coenzyme A via medium/long-chain acyl-CoA synthetases (ACSMs, ACSLs) and subsequently enter the mitochondria for oxidative catabolism by carnitine palmitoyltransferase 1 (CPT1). Unsaturated fatty acids are saturated before being oxidatively catabolized, and 2,4-dienoyl-CoA reductase 1 (DECR1) catalyzes the reduction of PUFAs in mitochondria. Inhibition of CPT1 promotes ferroptosis induced by RLS3 (Kagan et al., 2017), and knockdown of DECR1 promotes ferroptosis in prostate cancer cells (Blomme et al., 2020). These examples suggest that PUFA depletion by β-oxidation may reduce ferroptosis oxidation precursors and inhibit ferroptosis. The importance of *O*-GlcNAcylation for lipid synthesis in cancer cells is self-evident. However, there is a lack of direct evidence on whether *O*-GlcNAcylation regulates lipolysis in relation to ferroptosis.

Initially, free PUFAs were thought to be ferroptosis drivers. However, further studies have revealed that PUFAs must undergo esterification and be incorporated into the membrane lipid environment and oxidized by oxygenases (e.g., lipoxygenases, ALOXs) to transmit ferroptosis signals. PUFAs (i.e., arachidonic acid or adrenergic acids) are catalyzed by ACSL4 into fatty acyl-CoA. LPCAT3 then promotes the esterification of fatty acyl-CoA bound to membrane phospholipids (e.g., phosphatidylethanolamine, phosphatidylcholine) into PUFA-phospholipids (e.g., PUFA-phosphatidylethanolamine, PUFA-PE; PUFA-phosphatidylcholine, PUFA-PC). The inactivation of ACSL4 and LPCAT3 renders cells resistant to ferroptosis (Dixon et al., 2015; Doll et al., 2017). Zhang H. et al. (2022) showed that phosphorylation of ACSL4 on Thr328 promotes its activation, drives PUFA incorporation into phospholipids, and promotes ferroptosis (Zhang H. L. et al., 2022). For other members of the ACSLs, ACSL1 generates conjugated linoleates (e.g., α-eleostearic acid) capable of triggering ferroptosis (Beatty et al., 2021). ACSL3-activated exogenous monounsaturated fatty acids promote cellular resistance to ferroptosis by reducing the sensitivity of membrane lipids to oxidation (Magtanong et al., 2019), suggesting that ACSLs may play different roles in ferroptosis regulation in a substrate-dependent manner. Furthermore, ACSL4 can be *O*-GlcNAcylated in HCC cells, and its silencing can eliminate the promoting effect of *O*-GlcNAcylation on apoptosis (Wang Y. et al., 2020). However, there is a competitive relationship between phosphorylation and *O*-GlcNAcylation. Therefore, researchers should explore whether *O*-GlcNAcylation and phosphorylation crosstalk affects the sensitivity of ACSL4 to ferroptosis.

Finally, oxygenases, such as the ALOXs, trigger ferroptosis by oxidizing membrane phospholipids (Kagan et al., 2017). In addition, cytochrome P450 oxidoreductase (POR) contributes to lipid peroxidation during ferroptosis (Zou et al., 2020). Recent studies have found that the calcium-independent phospholipase, iPLA2β, releases oxidized PUFAs from the membrane phospholipids to inhibit P53-driven ferroptosis under ROS-induced stress (Chen D. et al., 2021), indirectly showing that oxidized PUFA tails do not promote ferroptosis after separation from membrane phospholipids. The 2022 review by Stockwell concluded that ferroptosis should not be considered a general type of oxidative stress but rather a lethal accumulation of membrane-localized lipid peroxides (Stockwell, 2022). In lipid metabolism, the production of PUFAs increases ferroptosis sensitivity. Most fatty acids are consumed by β-oxidation, which reduces the rate of lipid peroxidation. Lipid droplet formation protects the PUFAs from lipid oxidation during ferroptosis (Chen et al., 2021b). These data suggest that PUFA-mediated lipid synthesis and catabolism play important roles in regulating ferroptosis. The regulation of lipid metabolism by *O*-GlcNAcylation has been widely studied; however, the role of *O*-GlcNAcylation in lipid metabolism-mediated ferroptosis is still unclear. Thus, a better understanding of the effect of *O*-GlcNAcylation on lipid peroxidation will help researchers clarify the complex regulatory processes for ferroptosis.

## 7 Effects of *O*-GlcNAcylation on the morphology and function of subcellular organelles during ferroptosis

### 7.1 *O*-GlcNAcylation and mitochondria

For subcellular organelles, changes in the morphology or function of subcellular organelles may either facilitate or accompany ferroptosis. In recent years, the roles of different subcellular organelles in ferroptosis have been identified. Morphologically, mitochondria shrink in size, cristae are reduced, and the outer membranes are ruptured during ferroptosis (Dixon et al., 2012). Functionally, depletion of mitochondria or inhibition of the electron transport chain causes resistance to cystine starvation or erastin-induced ferroptosis (Gao et al., 2019). Voltage-dependent Anion channel 2 (VDAC2, responsible for the transport of ions and metabolites) of the outer mitochondrial membrane is a direct target of erastin, which causes mitochondrial dysfunction and massive oxide release via VDAC2 (Yagoda et al., 2007). In addition, mitochondria play an important role in iron metabolism. Iron in the LIP can be imported into mitochondria through channels in the outer mitochondrial membrane (e.g., SLC25A37 and SLC25A28) to synthesize heme and Fe-S clusters (Paradkar et al., 2009). Iron overload in the mitochondria can lead to mitochondrial autophagy (Li et al., 2018). Inhibition of the mitochondrial outer membrane protein CDGSH iron-containing domain-containing protein 1 (CISD1) increases mitochondrial iron accumulation and mitochondrial lipid peroxidation, promoting erastin-induced ferroptosis (Yuan et al., 2016). Ferroptosis may be initiated and amplified by damage to the mitochondrial morphology and function.

With the discovery of mitochondrial OGT (mOGT), the effect of *O*-GlcNAcylation on mitochondria has attracted widespread attention. Numerous publications have shown that *O*-GlcNAcylation is associated with mitochondrial dysfunction (Zhang X. et al., 2021; Xue et al., 2022). For example, Dontaine et al. (2022) found that acute elevation in mitochondrial *O*-GlcNAc modifications enhanced electron transport chain flux and complex I activity and decreased ROS release (Dontaine et al., 2022). Furthermore, many mitochondrial proteins involved in mitochondrial respiration, fatty acid metabolism, apoptosis, and other biological processes are substrates for mOGT (Jóźwiak et al., 2021). As mentioned above, Yu et al. found that inhibition of *O*-GlcNAcylation increased cellular ferritinophagy, and iron released from ferritinophagy was transported to the mitochondria. The investigators subsequently found that inhibition of *O*-GlcNAcylation increased mitochondrial fragmentation and autophagy, adding an additional source of iron for ferroptosis and making cells more sensitive to this mechanism of cell death (Yu et al., 2022). This study provides a different perspective on our understanding of ferroptosis in terms of the regulation of mitochondrial iron homeostasis by *O*-GlcNAcylation. It is unknown whether *O*-GlcNAcylation affects ferroptosis by regulating mitochondrial ROS.

### 7.2 *O*-GlcNAcylation and endoplasmic reticulum

Of the other subcellular organelles, the ER is worth mentioning. The efficacy of ferrostatin-1 (ferroptosis inhibitor) is derived from its ability to anchor to lipid membranes, thereby capturing free radical intermediates during lipid peroxidation and reducing lipid hydroperoxides. Gaschler et al. (2018) observed Ferrostatin-1 aggregates in lysosomes, mitochondria, and the ER using stimulated Raman scattering microscopy. Ferrostatin-1 accumulation in the ER may be critical for inhibiting ferroptosis (Gaschler et al., 2018). Consistent with this hypothesis, the fluorescent lipid peroxidation probe LiperFluo was mainly localized in the ER (Kagan et al., 2017). Because the ER contains more than half of the lipid bilayers of a cell and is the source of most membrane lipids in other organelles, it is not surprising that the ER may be an important site of lipid peroxidation. Another study showed that erastin could induce ALOX5 translocation to the nuclear membrane, suggesting that lipid peroxidation also occurs in that membrane (Yang et al., 2016). Thus, membrane damage may involve multiple subcellular organelles, and cells exhibiting loss of plasma membrane integrity may be in a late stage of ferroptosis (Stockwell, 2022). The ESCRT III may repair the final stage of plasma membrane damage but may also simply slow the rate of iron death (Pedrera et al., 2021). The epidermal growth factor (EGF) domain-specific *O*-GlcNAc transferase (EOGT) in the ER catalyzes proteins in the lumen of the ER, implying the presence of extracellular *O*-GlcNAc modifications (Sakaidani et al., 2011; Ogawa et al., 2015). Abnormal *O*-GlcNAcylation in the ER tubular lumen is associated with the development of some diseases (e.g., Adams-Oliver syndrome and Walker-Warburg syndrome) (Manzini et al., 2012; Shaheen et al., 2013; Cohen et al., 2014). Recent studies have found that the OGT inhibitor OSMI-1 can activate apoptosis by inducing ER stress (Lee et al., 2021). *O*-GlcNAcylation promotes the formation of Coat Protein Complex II (COPII) vesicles and accelerates the cis-transport of vesicles in the ER-Golgi network (Cho and Mook-Jung, 2018). These reports suggest that *O*-GlcNAcylation is associated with stress response and vesicle transport in the ER. However, it is unclear what types of proteins are modified by *O*-GlcNAc in the ER lumen or what the effect of *O*-GlcNAcylation is on ER function, especially whether *O*-GlcNAcylation regulates ER lipid metabolism.

## 8 Prospects

The biological processes and sensitivity of ferroptosis are highly dependent on the ROS defense system, iron metabolism and membrane lipid peroxidation metabolism (Figure 4). Studies of these three processes have identified many direct and indirect regulatory factors regulated by transcription, translation, epigenetics, and post-translational modifications (e.g., phosphorylation, methylation, and acetylation) that also play an indispensable role in ferroptosis (Wei et al., 2020). *O*-GlcNAcylation also regulates ferroptosis, adding to our understanding of ferroptosis from another perspective. However, the role of *O*-GlcNAcylation in ferroptosis is only beginning to be elucidated, so there are still many unknowns and questions. For example, physiological and pathological states of *O*-GlcNAc modifications have different regulatory effects on ferroptosis, and *O*-GlcNAc modifications in different cell types under the same or similar conditions may play different roles, indicating the complexity and refinement of *O*-GlcNAcylation in regulating ferroptosis.

Although ferroptosis is not equivalent to general ROS accumulation, the accumulation of ROS is necessary for ferroptosis. There is a crosstalk between ROS and *O*-GlcNAcylation in the antioxidant defense system. It is unclear whether *O*-GlcNAcylation plays a role in regulating ferroptosis through other defense systems in addition to the System Xc/GSH/GPX4 axis. It is worth mentioning that CoQ_10_H_2_ functions in the plasma membrane. Does the ER, the main site of lipids, also have other defense systems? Whether *O*-GlcNAcylation in the ER is involved in the ROS defense system is a question well worth exploring.

The significance of iron uptake, utilization, storage, and efflux in ferroptosis is self-evident. Recent findings only suggest that *O*-GlcNAcylation regulates ferroptosis sensitivity through iron metabolism; however, whether *O*-GlcNAcylation is a direct driver of iron metabolism under physiological or pathological conditions is unclear.

Whether *O*-GlcNAcylation occurs in key enzymes in the membrane lipid peroxidative metabolism pathway during the synthesis of PUFAs needs to be further characterized. Lipid droplet synthesis and catabolism and fatty acid β-oxidation appear to regulate PUFA content; however, free PUFAs may not be inherently ferroptosis-toxic. Therefore, PUFA oxidation (especially membrane PUFAs) should be the focus of attention. In contrast, the regulation of lipid substrate production and oxidative processes by *O*-GlcNAcylation currently lacks relevant evidence. It is worth noting that not all unsaturated fatty acids induce ferroptosis, and it is also worth considering whether *O*-GlcNAcylation regulates the production of different fatty acids (e.g., monounsaturated fatty acids).

Finally, whether other subcellular organelles (e.g., the Golgi) regulate ferroptosis needs to be explored. *O*-GlcNAcylation has been observed in the Golgi (Ogawa et al., 2015). In addition, neighboring cells might transmit ferroptosis signals. Does this signaling involve exosomes even though OGT is present within the exosome (Yuan et al., 2021)?

## 9 Conclusion

**FIGURE 5:**
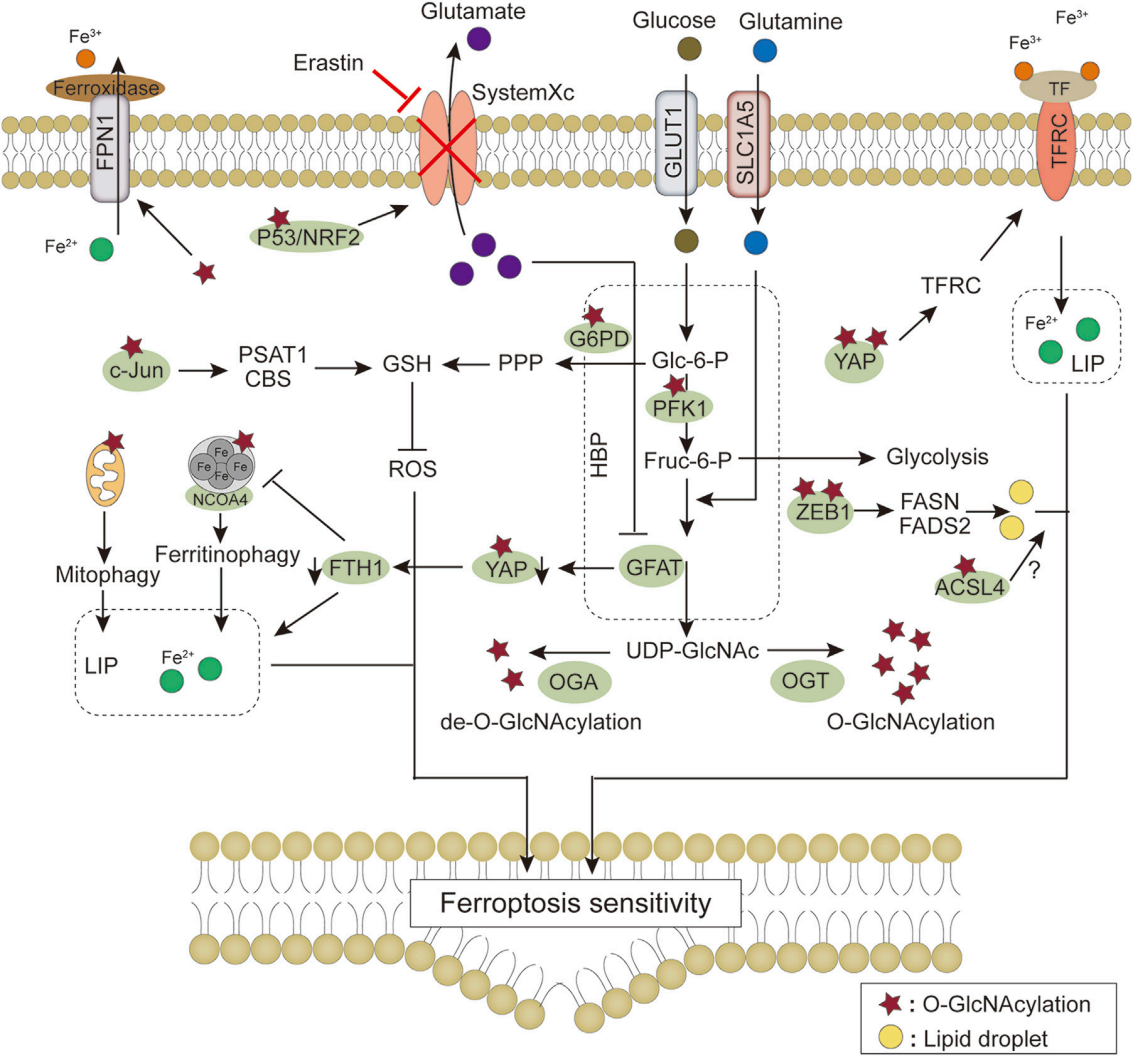
Modulation of ferroptosis signaling pathway by O-GlcNAc modification. HBP provides UDP-GlcNAc through glucose and glutamine, and regulation of O-GlcNAc modification homeostasis by OGT/OGA affects cellular sensitivity to ferroptosis through ROS production, iron metabolism, and fatty acid synthesis, respectively. Details of the molecular mechanism are provided in the main text. The star symbol represents O-GlcNAc modification, and the yellow orbs represent lipid droplets. Abbreviations: ACSL4, Long-chain fatty acid-CoA synthetase 4; CBS, cystathionine β synthase; FADS2, fatty acid desaturases 2; FASN, fatty acid synthetase; FPN1, ferroportin 1; Fruc-6-P, fructose-6-phosphate; FTH1, ferritin heavy chain 1; G6PD, glucose-6-phosphate dehydrogenase; GFAT, Glutamine-fructose-6-phosphate transaminase; Glc-6-P, Glucose-6-phosphate; GLUT1, Glucose transporter 1; GSH, glutathione; HBP, hexosamine biosynthetic pathway; LIP, labile iron pool; NRF2, Nuclear factor erythroid 2-related factor 2; OGA, *O*-GlcNAcase; OGT, *O*-GlcNAc transferase; PFK1, phosphofructokinase 1; PPP, pentose phosphate pathway; PSAT1, phosphoserine aminotransferase 1; SLC1A5, solute carrier family 1 member 5; TF, transferrin; TFRC, transferrin receptor; YAP, Yes-associated protein; ZEB1, zinc-finger E homeobox-binding 1.

**FIGURE 6:**
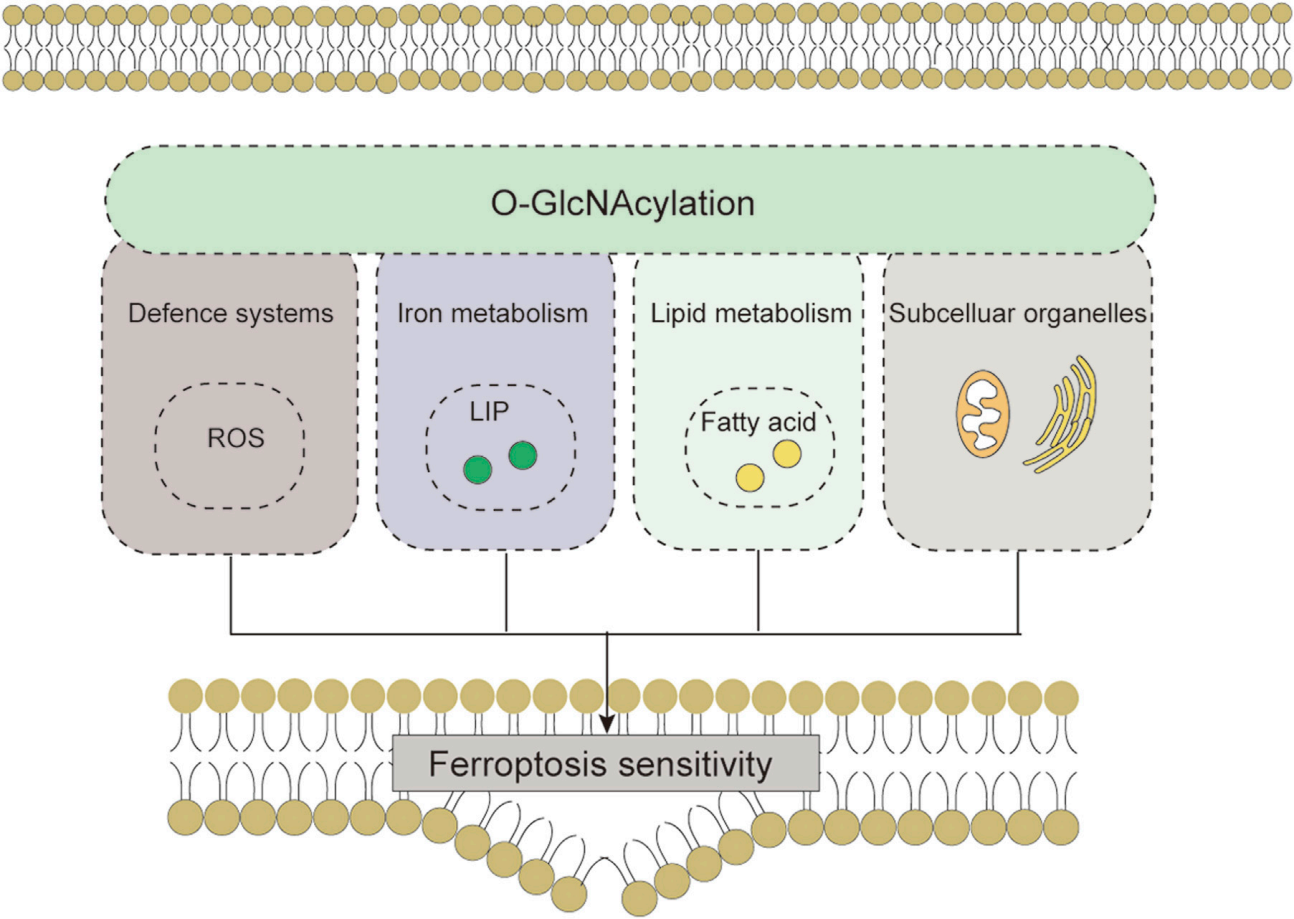
*O*-GlcNAcylation affects cellular sensitivity to ferroptosis through ROS biology, iron metabolism, lipid peroxidation and subcellular organelles. Abbreviations: ROS, reactive oxygen species; LIP, labile iron pool.

In summary, adaptive regulation by *O*-GlcNAcylation in response to stress perturbations plays an important role in regulating ferroptosis sensitivity via the antioxidant defense system-controlled ROS biology, iron metabolism, and membrane lipid peroxidation metabolism (Table 1). These three processes synergistically interact with each other. In lipid metabolism, *O*-GlcNAcylation is involved in regulating the process of lipid substrate production; in iron metabolism, *O*-GlcNAcylation affects the efficiency of Fe^2+^ utilization; the crosstalk with ROS biology reflects the role of *O*-GlcNAcylation in the defense process of lipid peroxidation damage (Figure 5). In addition, changes in the morphology and function of subcellular organelles regulated by *O*-GlcNAc modification may trigger and amplify ferroptosis (Figure 6). These current studies on the roles of *O*-GlcNAcylation in ferroptosis are only the prologue, and since one key cannot open all locks, we are not limited to starting with the above questions in the future. Exploring the environment-specific regulatory effects of *O*-GlcNAcylation is important for understanding the physiological and pathological mechanisms of ferroptosis.

**TABLE 1:** Effect of key *O*-GlcNAcylated proteins on ferroptosis.

Proteins	*O*-GlcNAcylation sites	Effect on its function	Mechanisms	Effect on ferroptosis	Sample types	Ref
c-Jun	Ser73	*O*-GlcNAcylation promotes protein expression, transcriptional activity and nuclear accumulation of c-Jun	*O*-GlcNAcylation of c-Jun stimulates GSH synthesis and reduces ROS accumulation	*O*-GlcNAcylation of c-Jun inhibits ferroptosis	Liver cancer	Chen et al. (2019)
YAP	Thr241	*O*-GlcNAcylation antagonizes Ser127 phosphorylation to inhibit the degradation of YAP	Reduced *O*-GlcNAcylation inhibit the transcription of FTH1 by YAP, resulting in elevated LIP	Decreased *O*-GlcNAcylation of YAP increased ferroptosis sensitivity	Lung adenocarcinoma	Zhang et al. (2021a)
YAP	Thr241	*O*-GlcNAcylation enhances and stabilizes the expression of YAP	*O*-GlcNAcylation of YAP increases TFRC transcription and leads to elevated Fe^2+^ concentration	Elevated YAP *O*-GlcNAcylation increases ferroptosis sensitivity	hepatocellular carcinoma	Zhu et al. (2021)
ZEB1	Ser555	*O*-GlcNAcylation enhances the stability and nuclear translocation of ZEB1	*O*-GlcNAcylation of ZEB1 promotes the transcriptional activity of adipogenesis-related genes FASN and FADS2, leading to increased synthesis of PUFAs	*O*-GlcNAcylation of ZEB1 promotes ferroptosis in mesenchymal pancreatic cancer cells	Mesenchymal pancreatic cancer cells	Wang et al. (2022)
FTH	Ser179	De-*O*-GlcNAcylation of FTH promoted the degradation of FTH	De-*O*-GlcNAcylation of FTH increases the interaction with NCOA4 and promotes ferritinophagy, leading to elevated LIP	Inhibition of *O*-GlcNAcylation of FTH activates ferroptosis	U2OS cells, HUVEC and HT1080 cells	Yu et al. (2022)
