# Supplementary material for: Emerging field: O-GlcNAcylation in ferroptosis
Source: Front Mol Biosci. 2023 May 11;10:1203269. doi: 10.3389/fmolb.2023.1203269 (PMC10213749; doi:10.3389/fmolb.2023.1203269)
Supplement: Supplementary file 1 [file Table1.DOCX]

Table 1. Effect of key *O*-GlcNAcylated proteins on ferroptosis

| Proteins | *O*-GlcNAcylation sites | Effect on its function | Mechanisms | Effect on ferroptosis | Sample Types | Ref |
| --- | --- | --- | --- | --- | --- | --- |
| c-Jun | Ser73 | *O*-GlcNAcylation promotes protein expression, transcriptional activity and nuclear accumulation of c-Jun | *O*-GlcNAcylation of c-Jun stimulates GSH synthesis and reduces ROS accumulation | *O*-GlcNAcylation of c-Jun inhibits ferroptosis | Liver cancer | (Chen et al., 2019) |
| YAP | Thr241 | *O*-GlcNAcylation antagonizes Ser127 phosphorylation to inhibit the degradation of YAP | Reduced *O*-GlcNAcylation inhibit the transcription of FTH1 by YAP, resulting in elevated LIP | Decreased *O*-GlcNAcylation of YAP increased ferroptosis sensitivity | Lung adenocarcinoma | (Zhang et al., 2021) |
| YAP | Thr241 | *O*-GlcNAcylation enhances and stabilizes the expression of YAP | *O*-GlcNAcylation of YAP increases TFRC transcription and leads to elevated Fe^2+^ concentration | Elevated YAP *O*-GlcNAcylation increases ferroptosis sensitivity | hepatocellular carcinoma | (Zhu et al., 2021) |
| ZEB1 | Ser555 | *O*-GlcNAcylation enhances the stability and nuclear translocation of ZEB1 | *O*-GlcNAcylation of ZEB1 promotes the transcriptional activity of adipogenesis-related genes FASN and FADS2, leading to increased synthesis of PUFAs | *O*-GlcNAcylation of ZEB1 promotes ferroptosis in mesenchymal pancreatic cancer cells | Mesenchymal pancreatic cancer cells | (Wang et al., 2022) |
| FTH | Ser179 | De-*O*-GlcNAcylation of FTH promoted the degradation of FTH | De-*O*-GlcNAcylation of FTH increases the interaction with NCOA4 and promotes ferritinophagy, leading to elevated LIP | Inhibition of *O*-GlcNAcylation of FTH activates ferroptosis | U2OS cells, HUVEC and HT1080 cells | (Yu et al., 2022) |
